# Seeing beyond political affiliations: The mediating role of perceived moral foundations on the partisan similarity-liking effect

**DOI:** 10.1371/journal.pone.0202101

**Published:** 2018-08-29

**Authors:** Kathryn Bruchmann, Birgit Koopmann-Holm, Aaron Scherer

**Affiliations:** 1 Department of Psychology, Santa Clara University, Santa Clara, CA, United States of America; 2 Department of Internal Medicine, University of Iowa, Iowa City, IA, United States of America; Fordham University, UNITED STATES

## Abstract

Decades of research have demonstrated that we like people who are more similar to us. The present research tested a potential mechanism for this similarity-liking effect in the domain of politics: the stereotype that people’s political orientation reflects their morals. People believe that Democrats are more likely to endorse individualizing morals like fairness and Republicans are more likely to endorse binding morals like obedience to authority. Prior to the 2016 election, American participants (N = 314) viewed an ostensible Facebook profile that shared an article endorsing conservative ideals (pro-Trump or pro-Republican), or liberal ideals (pro-Clinton or pro-Democrat). Participants rated the favorability of the profile-owner, and completed the Moral Foundations Questionnaire for the profile-owner and themselves. As predicted, participants liked the profile-owner more when they shared political beliefs, and used political stereotypes to infer the moral foundations of the profile-owner. Additionally, the perceived moral foundation endorsement of the profile owner differentially mediated the relationship between the ideology and evaluations of the profile owner based on the party affiliation of the participant: perceived individualizing foundations mediated the relationship for Democratic participants and perceived binding foundations mediated the relationship for Republican participants. In other words, people liked their in-group members more because they thought that the profile-owner endorsed a specific type of morals. In Study 2 (N = 486), we ruled out the potential explanation that any political stereotype can account for the similarity-liking effect, replicating the results of Study 1 even when controlling for perceptions of other personality differences. Taken together, these studies highlight that there may be something unique about the perceived type of morality of political in-group and out-group members that may be contributing to the similarity-liking effect in politics.

## Introduction

The 2016 presidential race was historic in part because of the intense division of the political landscape[1]. The election featured the most disliked presidential candidates in history [2], and the partisan divide was the greatest in two decades. According to the Pew Research Center [1], more than half of Democrats and Republicans reported not just disliking each other, but actually being afraid of each other. This partisan divide was especially present on social media platforms like Facebook, where evidence suggests that people are more likely to be “friends” with politically like-minded individuals [3] and be motivated to avoid political outgroup members’ opinions [4]. Consequently, social media newsfeeds look remarkably different for Democrat or Republican Facebook users [5] both in the attitudes and opinions that are shared, but also in what news stories are shared. Previous research has demonstrated that people tend to like or want to engage with people similar to them [6, 7]; the present work investigates how the stereotypes that people hold about Democrats and Republicans—particularly about their moral beliefs—might account for this similarity-liking effect in politics.

### Similarity-liking effect

Initially labeled by Byrne [6, 7] as the “Attraction Paradigm”, there has now been decades of research supporting the idea that we like people who are similar to us [8, 9, 10]. This similarity-liking effect emerges regardless of whether the similarity is based on something significant, like important attitudes [11], or something irrelevant, like sharing birthdays [12]. People particularly like others who change their attitudes to become more aligned with their own [13]. There is evidence that these effects even happen at a neurological level; research found that the same brain region is activated when people are mentalizing about themselves or similar (but not dissimilar) others [14]. Other work suggests that this similarity-liking effect could also work in reverse (i.e., a liking-similarity effect) such that likeable people are considered to be more similar to the self than unlikeable people [15].

Similarity-liking effects are particularly strong for political attitudes [16, 17]. In addition to liking people more when they share political attitudes [16], people favor their political in-group, influencing everything from their willingness to give money to or share an office with others [18] to the recollection of a political candidate’s face [19]. People are also more likely than chance to be in romantic relationships with politically similar others [20], suggesting politically-driven assortative mating. People can categorize the political affiliation of others even without conscious awareness, suggesting it is an important aspect of how we form impressions of others [21]. Another example of this is that people may even be drawn to politically similar people on an olfactory level; people prefer the body odors of people in the same political party over people from the opposing party [22]. People also tend to *dislike* others who hold *different* values, beliefs, or attitudes [6, 23, 10]; changing an initially positive impression of others when discovering political dissimilarities [24]. Moreover, people perceive those who disagree on political issues to be more dissimilar to them than they actually are [17, 25].

But what drives political homophily? Do we like people more simply because of a shared group membership? We know from research using the minimal groups methodology [26] that even small, irrelevant distinctions between groups can lead to preferential treatment for ingroup members, and that this bias can increase when the group divisions are more pronounced [27]. Because the divide between political parties is perceived to be large—in many cases bigger than it actually is [28, 29]—more ingroup favoritism and outgroup bias is expected. However, some argue that the distinction between ingroup and outgroup members may just be an extension of the similarity-liking effect; in other words, one path to determining group membership itself is through assessing similarity to another person [30].

The present research argues that political group membership may signal other aspects of people that lead to the similarity-liking effect. Namely, we posit that people might use stereotypes about a person’s politics to guide subsequent judgments. While we know that stereotypes generally guide judgments and behavior [31], stereotypes about political groups could have arguably *larger* downstream consequences since there are fewer taboos against holding them, expressing them, or acting on them unlike stereotypes about racial, gender, or religious groups [32]. Specifically, we predict that stereotypes about the morals of different political parties will in part explain the similarity-liking effect.

#### Political stereotypes

Not surprisingly, people hold political stereotypes about the attitudes specific party members will have towards issues [17, 25, 33, 34, 35, 36]. However, people even make assumptions about personality based on political party [37, 38]. Research suggests that people perceive Democrats to be warmer and Republicans to be more competent, with these stereotypes being particularly pronounced for ingroup members [39]. Other research has demonstrated that there are stereotypes about the physical appearance of Democrats and Republicans [40, 37, 41, 42]. Research utilizing a reverse correlation methodology suggests that people’s mentalizations of liberals’ faces are happier and less angry than conservatives’ faces [41]. There is some evidence that this might represent actual differences in appearance. Multiple studies have demonstrated that people can identify a person’s political affiliation at greater than chance level just by looking at a photograph [37, 40, 42]. Researchers suggest that these judgments may be based on stereotypes that Republicans express more dominance and facial maturity, whereas Democrats express more likeability and trustworthiness [37]. Additionally, political candidates’ facial structure can predict whether or not they are elected; in the absence of party information, Republicans were shown to prefer “Republican looking” candidates, though Democrats did not demonstrate a preference based on candidates’ facial differences [40].

People also hold political stereotypes about psychological differences between liberals and conservatives. Some research suggests that conservativism is a form of motivated social cognition given the correlations between political conservatism and traits like fear of threats or death, intolerance of ambiguity, and needs for structure and closure [43]. Based on these findings, Scherer and colleagues [38] examined whether *stereotypes* about these political differences exist. Their results demonstrate that people believe that conservatives are more likely than liberals to be motivated to reduce uncertainty (Need for Cognitive Closure)[44], to believe the world to be dangerous (Belief in a Dangerous World) [45], to prefer a hierarchical society over equality (Social Dominance Orientation) [46], and to prefer existing social structures over ideas of social change (System Justification) [47]. While the patterns of these stereotypes reflect actual differences between liberals and conservatives, people exaggerate these differences [38].

#### Moral foundations theory

Critically for the present research, people hold political stereotypes that exaggerate actual differences in the types of morals that conservatives and liberals [34, 48]. According to Moral Foundations Theory [49] liberals are more likely to endorse *individualizing foundations*, which focus on individuals’ rights through *preventing harm* towards others and promoting *fairness*, while conservatives tend to be more likely to endorse *binding foundations*, which are factors that bind groups together through respecting *authority*, loyalty to *in-group* members, and physical or spiritual *purity*. People often cite these moral features as the reason for their political beliefs; with conservatives invoking binding foundations and liberals invoking individualizing foundations when defending their politics [50]. Conservatives and liberals even narrate their lives differently based on moral foundations; content analysis demonstrated that conservatives are more likely to describe lessons from strict moral authorities, whereas liberals are more likely to describe lessons regarding empathy for others [51].

Endorsement of these different moral foundations predicts partisan differences on political issues, such as support for same-sex marriage [52] or stem-cell research [53]. Moral foundations also influence behaviors such as donations to causes [54, 55]; voting [56]; or eco-friendly behaviors like using energy-efficient lightbulbs [57].

Based on these findings that ‘typical’ liberals or conservatives are perceived as having different moral foundations, we predict that when learning a person’s political orientation, people will make judgments about that person’s moral beliefs. We also predict that the perceptions of a person’s moral foundations should predict the overall impression that is formed of the person.

In general, moral people are considered to be more likeable [58]. Perceived morality predicts how favorably a person or group of people is viewed even beyond that person’s general warmth or competence [59, 60, 61]. Perhaps because of this, when gathering information in order to form an impression, people are more interested in gathering morality information than sociability information [62]. Additionally, positive traits like sociability and competence only lead to favorable impressions for moral (vs. immoral) people [63]. Additionally, recent research found that we tend to dehumanize people who are perceived as *immoral* and assume that they experience less social pain [64]. As a result, perceiving others as more moral predicts more positive behavioral intentions towards them [59, 65].

On a group level, people are more likely to identify with ingroups that are moral whether or not they are competent or sociable [66], are more likely to adhere to morality based ingroup norms [67], and are more willing to work with an ingroup towards morality based goals even if competence could suffer [68]. Additionally, when we perceive an outgroup to be less moral, we require less evidence to confirm their immorality [69]. This intergroup moral perception can be particularly important for politics; during the 2016 election season, media outlets focused the majority of their coverage on the (im)morality of the two presidential candidates [70]. Because morality is so important to impression formation, and so tightly coupled with political orientation, we argue that overall impressions of others formed on the basis of political affiliation are likely to be based in perceptions of morality. Additionally, we hypothesize that the two major groups of moral foundations (individualizing and binding) will differentially explain the similarity-liking effect for Democrats and Republicans given their different emphases by these two groups.

### Present research

The present research tested how learning someone’s political orientation from a Facebook post can influence the impression that is formed of the profile-owner and the consequences of these impressions. While scrolling past posts in a newsfeed might not provide much detail, there is evidence that small snippets of information can lead to accurate judgments[71]. For example, after seeing an online post for only five seconds, people made spontaneous trait inferences about the target [72]. In the present study, we were specifically interested in the types of impressions people form when seeing the profile of a person with political posts on Facebook.

Across two studies, participants viewed a Facebook profile that shared a political article indicating that the profile-owner was a Democrat or Republican. After viewing the Facebook profile, participants rated the profile-owner’s moral foundations and general favorability. We hypothesized that people would base their judgments of the profile-owner on political stereotypes; profiles sharing liberal information would be perceived as endorsing more individualizing foundations and less binding foundations than profiles sharing conservative information. Consistent with previous research on the similarity-liking effect, we predicted that profile-owners would be rated more favorably when they shared political affiliation with participants; however, we expected that these effects would be *due to the perception of moral foundations*. In other words, we expected that perceptions of the profile-owner’s moral foundations would mediate the similarity-liking effect. Furthermore, we predicted that the participants’ political beliefs would moderate this mediation such that different morals should matter for Democrats and Republicans. Specifically, we predicted that for Democrats, perceptions of individualizing foundations would be associated with greater favorability of the profile-owner while for Republicans, perceptions of binding foundations would be associated with greater favorability. In Study 2, we examined whether these mediators would hold even when controlling for other political stereotypes linked to personality traits associated with motivated social cognition.

## Study 1

### Method

#### Participants and design

Three months before the 2016 presidential election, 524 American MTurk workers (57.8% female, 23% nonwhite; *M*_age_ = 35 years, *SD* = 11.7 years) participated in a study described as assessing “social media impressions”. The study was approved by the Santa Clara University IRB (application #16-05-810), and participants provided consent via online survey.

#### Materials and procedure

Participants viewed an image of a Facebook profile that was constructed by the research team. Across all conditions, the profile featured a photo of a white man and woman, several group photos, 749 friends, and general posts about vacation photos, TV shows, etc. All posts were liked and commented on by “friends”. All identifying information was hidden. In all conditions, the profile’s most recent shared article was political in nature; it was either Republican (pro-Trump, or pro-Republican) or Democratic in nature (pro-Clinton, or pro-Democrat) with the caption “I couldn’t agree more”. A control condition with no political affiliation information was included for the purposes of a different research question; as the results are not pertinent here, this condition will not be discussed. See S1 for the associated ANOVA analyses for this condition.

After viewing the profile, participants rated the likeability and intelligence of the target (1 = *extremely unlikeable/unintelligent*, 7 = *extremely likeable/intelligent*) and indicated how likely they would become friends with the target (1 = *extremely unlikely*, 7 = *extremely likely*). As part of the cover story, participants also listed what they remembered from the Facebook profile and described other characteristics or traits that they believe the profile-owner might have.

Participants then completed a version of the 30-item Moral Foundations Questionnaire (MFQ) [49] which was edited so that the questions were about the profile-owner. The MFQ assesses each of the five moral foundations in two ways. First, participants indicated the relevance of each moral foundation for different concerns, specifying the extent to which they thought the profile-owner would think a factor linked to the moral foundations was relevant for deciding whether something was right or wrong (0 = *not at all relevant*, 5 = *extremely relevant*). Examples include whether or not someone “suffered emotionally” (*harm*) or “violated standards of purity and decency” (*purity*). Second, in the “judgments” portion, participants indicated to what extent they thought the profile-owner would agree with statements linked to moral foundations (0 = *strongly disagree*, 5 = *strongly agree*). Examples include “justice is the most important requirement for a society” (*fairness*) or “respect for authority is something all children need to learn” (*authority*).

Participants then completed the MFQ for themselves and provided demographic information. Participants indicated whether they think of themselves as Democrats, Republicans, or Independents/Other, and who they were planning on voting for in the 2016 election.

Participants were then probed for suspicion about the study and then were asked to recall the political affiliation of the target person in the profile before being debriefed.

### Results

Data for both studies can be found here: https://figshare.com/articles/Seeing_beyond_political_affiliations_The_mediating_role_of_perceived_moral_foundations_on_the_partisan_similarity-liking_effect/6972392. Participants self-identified as Democrats (63.51%), Republicans (31.75%) and Other (24.17%). Because we were interested in how political similarity could influence perceptions, we excluded the people who did not identify as Republican or Democrat from our analyses. Of the remaining participants (N = 314), 76.9% of Democrats planned on voting for Hillary Clinton and 75% of Republicans planned on voting for Donald Trump. Participants’ political affiliations did not differ by condition, *F*<1.

#### Manipulation check

To check our Facebook manipulation, we coded participants’ responses to what political affiliation the profile-owner had. Liberal responses (e.g., Clinton or Democrat) were coded as -1, no answer or “unsure” was coded as 0, and conservative responses (e.g., Trump or Republican) were coded as 1. An ANOVA revealed a main effect of Facebook condition confirming that our manipulation worked, *F*(1, 310) = 620.22, *p* < .001, η_p_^2^ = .667. The Democratic profile-owners (*M* = -0.78, *SD* = 0.44) were rated as more liberal than the Republican profile-owners (*M* = 0.70, *SD* = 0.55).

### Preliminary analyses

In order to test whether our sample showed the typical response patterns for endorsement of moral foundations, the self-MFQ was scored; composite scores were calculated for individualizing foundations (harm and fairness, α = .82) and binding foundations (loyalty, authority, and purity, α = .87). Participants’ political party predicted responses to both individualizing foundations, *F*(1, 310) = 25.03, *p* < .001, η_p_^2^ = .075, and binding foundations, *F*(1, 310) = 50.97, *p* < .001, η_p_^2^ = .141). Consistent with previous research, Democrats (*M* = 29.19, *SD* = 4.34) were more likely to endorse individualizing foundations than Republicans (*M* = 26.56, *SD* = 4.88), and Republicans (*M* = 25.34, *SD* = 4.91) were more likely to endorse binding foundations than Democrats (*M* = 20.52, *SD* = 5.94). Using paired samples *t*-tests, we found that both Democrats, *t*(205) = 18.64, *p* < .001, and Republicans, *t*(107) = 2.66, *p* = .009 were more likely to endorse individualizing foundations than binding foundations. Participants’ self-ratings of binding foundations were not different across Facebook profile conditions, *F*<1. Unexpectedly, self-ratings of individualizing foundations were different, *F*(1,310) = 9.83, p = .002, η_p_^2^ = .031; participants who saw the Republican profile-owner were more likely to endorse individualizing foundations (*M* = 29.02, *SD* = 4.00) than participants who saw the Democrat profile-owner (*M* = 27.60, *SD* = 5.18).

#### Profile-owner’s moral foundations

The MFQ was also scored for the profile-owner ratings and composite scores were calculated for individualizing foundations (α = .91) and binding foundations (α = .87). See Table 1 for all profile-owner ratings.

**Table 1:** Mean ratings of profile-owner by Facebook profile condition and participant political party.

	Participant Political Party	Facebook Profile Condition	
Measure		Democrat	Republican
Individualizing Foundations	Democrat	26.17 (5.76)	22.44 (6.67)
	Republican	26.06 (4.78)	25.86 (4.76)
	Total	26.13 (5.44)	23.67 (6.25)
Binding Foundations	Democrat	20.85 (4.79)	23.15 (4.85)
	Republican	21.47 (5.85)	23.48 (4.93)
	Total	21.06 (5.16)	23.27 (4.86)
Favorability	Democrat	5.25 (0.97)	4.06 (1.36)
	Republican	4.49 (1.41)	5.18 (1.13)
	Total	5.00 (1.19)	4.46 (1.39)

*Note*. Standard deviations appear in parentheses.

Ratings of the profile-owner’s individualizing foundations were subjected to a 2 (Facebook profile: Democrat, Republican) x 2 (participant political party: Democrat, Republican) ANOVA. The effect of participant political party was significant, *F(*1, 310) = 5.85, *p* = .016, η_p_^2^ = .019 such that Democrats rated the profile-owners (*M* = 24.42, *SD* = 6.46) lower in individualizing foundations than Republicans (*M* = 25.96, *SD* = 4.75). A main effect of Facebook condition also emerged, *F*(1, 310) = 8.20, *p* = .004, η_p_^2^ = .026. Consistent with previous research (Graham et al., 2012), the Democratic profile-owners was rated as more likely to endorse individualizing foundations than the Republican profile-. This main effect was qualified by a participant political party x Facebook condition interaction, *F*(1, 310) = 6.65 *p* = .01, η_p_^2^ = .021. For Democrats, Republican profile-owners were seen as less likely to endorse individualizing foundations than Democratic profile-owners (*p* < .001, *d* = -0.60). For Republicans, there was no difference in the ratings of the profile-owner’s binding foundations across Facebook profile condition.

Ratings of the profile-owner’s binding foundations were subjected to a 2 (Facebook profile: Democrat, Republican) x 2 (participant political party: Republican, Democrat) ANOVA. A main effect of Facebook condition emerged, *F*(1, 310) = 13.05, *p* < .001, η_p_^2^ = .04. As predicted, the Republican profile-owners were considered more likely to endorse binding foundations than the Democratic profile-owners. There was not a significant main effect of participant political party or a significant interaction, *F*s<1.

#### Favorability of profile-owner

The participants’ ratings of the target’s likeability, intelligence, and the likelihood of becoming friends were averaged to create a favorability composite (α = .88) where higher numbers indicate greater favorability. A Facebook condition x participant political party ANOVA revealed only a significant interaction, *F*(1, 310) = 42.89, *p* < .001, η_p_^2^ = .122, As predicted by the similarity-liking effect, Democrats rated the Democratic profile-owners more favorably than the Republican profile-owners (*p* < .001, *d* = 1.02), and Republicans rated the Republican profile-owners more favorably than the Democratic profile-owners (*p* = .003, *d* = .70).

#### Do moral foundations explain political similarity-liking effects?

**Fig 1:**
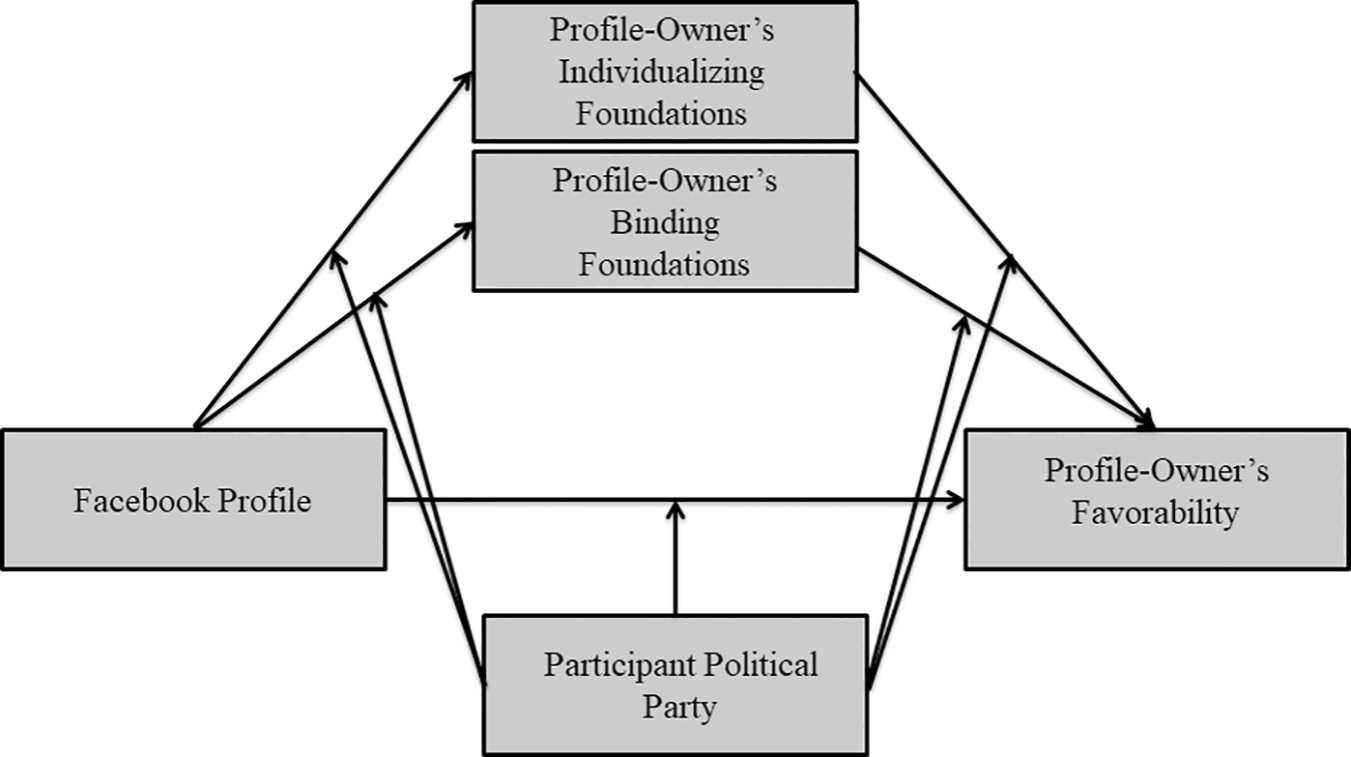
Predicted model of moderated mediation.

We hypothesized that that ratings of the profile-owner’s moral foundations would mediate the similarity-liking effect. More specifically, we hypothesized that *different* moral foundations would mediate the effect of Facebook Profile on the favorability of the profile-owner for Republicans and Democrats (see Fig 1): we predicted that Democrats would like the Democratic profile-owner more because they would perceive him to endorse more *individualizing* foundations, and that Republicans would like the Republican profile-owner more when because they would perceive him to endorse more *binding* foundations. To test our hypothesis of moderated mediation, we used Hayes’ PROCESS Macro for SPSS[73]. First, we ran a moderated mediation model (PROCESS model 59) to examine whether the participant political party moderated the mediational role of binding and individualizing foundations on the relationship between the Facebook profile condition and the favorability of the profile-owner. Facebook profile (1 = Democratic profile, -1 = Republican profile) was entered as the independent variable (X), the composite favorability rating of the profile-owner was entered as the outcome variable (Y), the ratings of the profile-owner’s binding foundations and individualizing foundations were entered as the mediators (M_1_ and M_2_), and participant political party (1 = Democrat, -1 = Republican) was entered as moderator (W). The conditional direct effect of Facebook Profile on the Favorability Rating of the profile-owner was significant for Republican participants, *B*_*c’*_ = -.30, *SE* = .11, *t*(306) = -2.68, *p* < .01, and for Democrat participants, and *B*_*c’*_ = .52, *SE* = .09, *t*(306) = 5.94, *p* < .001; as described above in the ANOVA analyses, Republican participants rated the Republican profile-owner more favorably, and Democrat participants rated the Democratic profile-owners more favorably.

As predicted, the conditional indirect effect of Facebook profile condition on the favorability of the profile-owner through ratings of the profile-owner’s individualizing moral foundations was significant only for Democrats (95% CI: 0.05 to 0.21), but not for Republicans (95% CI: -0.04 to 0.08). The index of moderated mediation was significant for individualizing foundations (95%CI: 0.02 to 0.22) using 5,000 bootstrap samples. In other words, Democrats liked the Democratic profile-owners more in part because they perceived him to endorse more individualizing foundations, but perceptions of the profile-owner’s individualizing foundations did not explain the Republican’s favorability ratings of the profile-owner.

Unexpectedly, the conditional indirect effect of Facebook Profile on the favorability of the profile-owner through ratings of the profile-owner’s binding foundations was not significant for Republicans (95% CI: -0.18 to .01), or Democrats (95%CI: -0.09 to 0.00). The index of moderated mediation was not significant for binding moral foundations (95% CI: -0.06 and 0.14) with 5,000 bootstrap samples. In other words, perceptions of the profile-owner’s endorsement of binding foundations did not explain the similarity-liking effect for Democrats or Republicans.

**Fig 2:**
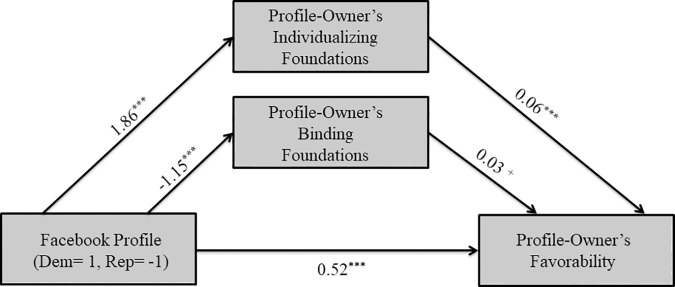
Mediational model for Democrat participants in Study 1. Solid lines indicate paths significant at *p*≤.10, while dashed lines indicate non-significant paths. ^+^ *p*≤.10 *p≤.05 **p≤.01 ***p≤.001.

**Fig 3:**
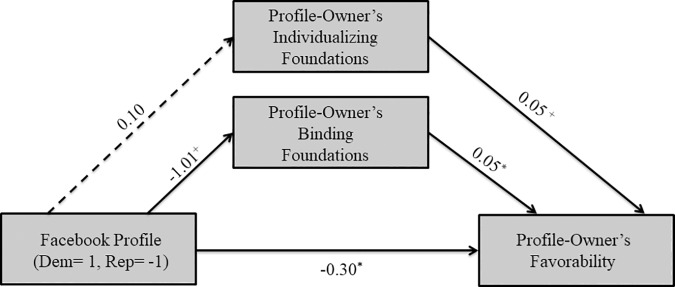
Mediational model for Republican participants in Study 1. Solid lines indicate paths significant at *p*≤.10, while dashed lines indicate non-significant paths. ^+^ *p*≤.10 *p≤.05 **p≤.01 ***p≤.001.

To examine the nature of the moderating effects, we ran a simple mediation model (PROCESS Model 4) for Democrats (see Fig 2) and Republicans (See Fig 3) separately. As hypothesized, the Democratic profile-owner was associated with greater perceived individualizing foundations (*B* = 1.86, *SE* = .43, *t*(204) = 4.30, *p* < .001) and lower perceived binding foundations (*B* = -1.15, *SE* = .34, *t*(204) = -3.43, *p* < .001) by Democratic participants. Furthermore, greater perceived individualizing foundations (*B* = .06, *SE* = .01, *t*(202) = 4.86, *p* < .001) and the Democratic profile-owners (*B* = .52, *SE* = .08, *t*(202) = 6.18, *p* < .001) were associated with more favorability. There was a marginally significant positive association between perceived binding foundations and favorability (*B* = .03, *SE* = .02, *t*(202) = 1.77, *p* = .08). In sum, Democrats liked the Democratic profile-owner more than the Republican profile-owner in part because they perceived him to endorse more individualizing foundations.

For Republican participants, there was a marginally significant positive association between the Republican profile-owner (coded as -1) and binding foundations (*B* = -1.01, *SE* = .52, *t*(106) = -1.93, *p* = .06), and no significant association with perceived individualizing foundations (*B* = .10, *SE* = .46, *t*(106) = .21, *p* = .83) higher., As predicted, greater perceived binding foundations (*B* = .05, *SE* = .02, *t*(104) = 2.04, *p* < .05) and the Republican profile-owner (*B* = -.30, *SE* = .12, *t*(104) = -2.50, *p* < .05) were associated with more favorability. Interestingly, perceived individualizing foundations were also marginally significantly associated with more favorability (*B* = .05, *SE* = .03, *t*(104) = 1.88, *p* = .06). In sum, we found suggestive evidence for our hypothesis that Republicans liked the Republican profile-owner more than the Democratic profile-owner in part because they perceived him to endorse more binding foundations.

### Discussion

As expected, we found strong evidence for the similarity-liking effect. Participants liked the profile-owners more when they were political in-group members versus political out-group members. Importantly, this study provided the first evidence of a mechanism behind political homophily: moral foundations. We demonstrated that people use political stereotypes about moral foundations when learning a person’s political beliefs. Consistent with previous research, participants believed that Democrats preferentially endorse individualizing foundations and that Republicans preferentially endorse binding foundations. Interestingly, these effects were stronger among Democrat than Republican participants, which may make sense given that Democrats tend to exhibit greater stereotype exaggeration compared to Republicans (e.g., Graham et al., 2012; Scherer et al., 2015). Perceptions of these morals, in turn, predicted how favorably the profile-owner was viewed overall. As predicted, for Democrat participants, individualizing foundations were a stronger predictor of a favorable impression than binding foundations, whereas the opposite was true for Republican participants. It is already widely established that morals are an important aspect of impression formation (e.g., Goodwin et al., 2014). However, our study is the first to demonstrate that *different kinds* of morals matter for *different kinds* of people: For Democrat participants, individualizing foundations partially explained the similarity-liking effect, whereas for Republican participants, we found marginally significant evidence for our prediction that binding foundations can partly explain this effect.

## Study 2

Study 1 provided initial evidence of a mechanism (i.e., mediator) behind the political similarity-liking effect. Study 1 suggests that the nature of this mechanism depends on the political party of the participants (i.e., moderated mediation). The aim of Study 2 was to determine whether the moderated mediation effects of the political moral foundations stereotypes on the similarity-liking effect persist when controlling for other political stereotypes. To do so, participants rated the Facebook profile-owner on other psychological measures in which political stereotypes have been demonstrated. Previous research [38] has demonstrated that there are stereotypes (that are exaggerations of true political differences) that Republicans are likely to be higher than Democrats in the traits of Need for Cognitive Closure (NFCC) [44], Belief in a Dangerous World (BDW) [45], Social Dominance Orientation (SDO) [46], and System Justification (SJ) [47]. Because of the unique importance of morality in impression formation [61] we hypothesized that political stereotypes about moral foundations would still partially explain the similarity-liking effect even when controlling for political stereotypes about traits linked to motivated social cognition. Additionally, we improved upon Study 1 by recruiting a larger sample of Republicans. This was important to examine whether the marginally significant findings for Republicans in Study 1 were a result of being underpowered to detect the mediating role of moral foundations among Republicans.

### Method

#### Participants and design

We recruited 520 American MTurk workers who were pre-screened as being politically liberal (N = 260) and conservative (N = 260) to participate in this study 15 months after the 2016 election. Participants then categorized themselves as Democrat (54.31%), Republican (41.15%), or Independent/Other (6.52%); as in Study 1, participants who did not identify as either Democrat or Republican were dropped from the analysis leaving a sample of 486 (65% female, 12.85% non-white; *M*_age_ = 40.88 years, *SD* = 12.73 years). Participants were randomly assigned to view one of two Facebook profiles: Democrat or Republican. The study was IRB approved and participants provided consent via online survey.

#### Materials and procedure

The procedures were largely the same as in Study 1. The pro-Republican and pro-Democrat profiles from Study 1 were used, but with updated timestamps on all posts, and more recent (post-election) articles shared with captions updated to say “I am proud to be a Democrat (Republican)”.

Participants completed the short form 20-item MFQ [49] from the perspective of the profile-owner. Additionally, participants rated the profile-owner on shortened versions of several other measures. First, participants completed the fifteen item short form of the NFCC scale [74] in which they indicated how much they thought the profile-owner would agree with statements such as “I don’t like situations that are uncertain” (1 = *strongly disagree*, 6 = *strongly agree*). Next, participants completed four items from the BDW Scale [45] in which they indicated how much they thought the profile-owner would agree with statements such as “Despite what one hears about ‘crime in the street’, there probably isn’t any more now than there ever has been (1 = *strongly disagree*, 6 = *strongly agree*). Then, in the short form 4 item- SDO scale [46], participants indicated how the profile-owner would feel towards statements like “If certain groups stayed in their place, we would have fewer problems” (1 = *very negative*, 7 = *very positive*). Finally, participants completed a shortened version of a measure of SJ [47] in which they reported how much they thought the profile-owner would agree with statements like “Society is set up so that people usually get what they deserve” (1 = *strongly disagree*, 9 = *strongly agree*).

Participants then indicated how likable and intelligent they thought the profile-owner was (1 = *extremely unlikable/unintelligent*, 7 = *extremely likable/intelligent*), and how negatively or positively they felt about the person in general (1 = *extremely negative*, 7 = *extremely positive*). They also rated how likely they would be to become friends with the person (1 = *extremely unlikely*, 7 = *extremely likely*), and how much they would want the profile-owner as a neighbor (1 = *not at all*, 7 = *very much*).

Finally, participants completed the individual difference measures for themselves before they were probed for suspicion and debriefed.

### Results

#### Preliminary analyses

The participants’ self-rated MFQ was scored and composite scores were calculated for individualizing foundations (harm and fairness, α = .78) and binding foundations (loyalty, authority, and purity, α = .85). Participants’ political party predicted responses to both individualizing foundations, *F*(1, 484) = 29.91, *p* < .001, η_p_^2^ = .058, and binding foundations, *F*(1, 484) = 150.99, *p* < .001, η_p_^2^ = .238. See Table 2 for means and standard deviations of all self-rated measures. Consistent with Study 1, Democrats were more likely to endorse individualizing foundations than Republicans and Republicans were more likely to endorse binding foundations than Democrats. Again, consistent with Study 1, both Democrats *t*(271) = 24.94, *p* < .001 and Republicans *t*(213) = 4.96, *p* < .001 were more likely to endorse individualizing foundations than binding. Participants’ self-ratings were marginally different across Facebook profile conditions for both individualizing foundations (*F*(1, 482) = 3.35, *p* = .068, η_p_^2^ = .007) and binding foundations (*F*(1, 482) = 3.47, *p* = .063, η_p_^2^ = .007) such that participants who compared to Republican profiles rated themselves as marginally higher in both binding foundations (*M* = 14.78, *SD* = 4.66) and individualizing foundations (*M* = 19.58, *SD* = 3.19) than participants who compared to Democrat profiles (*M* = 14.17, *SD* = 4.42; *M* = 19.07, *SD* = 3.24).

**Table 2:** Study 2 means of self-ratings, by participant political party.

Political Party	Individualizing	Binding	NFCC	BDW	SDO	SJ
Democrat	19.86 (2.92)	12.50 (4.24)	3.84, (0.92)	12.15 (4.86)	7.84 (4.25)	12.20 (5.61)
Republican	18.44 (3.50)	16.95 (3.63)	4.11 (0.96)	14.96 (5.02)	14.71 (5.86)	17.09 (5.31)

Note. Standard deviations are in parentheses.

The motivated social cognition measures were scored and subjected to Facebook condition x participant political party ANOVAs; Consistent with predictions, Republicans rated themselves higher in NFCC (α = .92, *F*(1, 482) = 9.20, *p* = .003, η_p_^2^ = .019), BDW (α = .83, *F*(1, 482) = 38.04, *p* < .001, η_p_^2^ = .073), SDO (α = .84, *F*(1, 482) = 223.14, *p* < .001, η_p_^2^ = .316), and SJ (α = .77, *F*(1, 482) = 96.10, *p* < .001, η_p_^2^ = .166) than Democrats. No effects of Facebook condition or interactions emerged.

#### Ratings of profile-owner

The profile-owner’s MFQ was scored and composite scores were calculated for individualizing foundations (harm and fairness, α = .91) and binding foundations (loyalty, authority, and purity, α = .89). See Table 3 for means and standard deviations of all ratings of the profile-owner.

**Table 3:** Study 2 mean ratings of profile-owner by Facebook condition and participants’ political party.

		Facebook Profile Condition	
Measure	Participant Party	Democrat	Republican
Individualizing Foundations	Democrat	18.78 (3.39)	14.75 (4.42)
	Republican	17.60 (3.69)	17.70 (3.54)
	Total	18.26 (3.57)	16.04 (4.31)
Binding Foundations	Democrat	13.67 (3.79)	17.06 (3.48)
	Republican	12.97 (3.99)	17.20 (3.64)
	Total	13.35 (3.88)	17.12 (3.54)
Need for Cognitive Closure	Democrat	3.45 (0.91)	4.15 (0.85)
	Republican	3.65 (0.84)	3.93 (1.01)
	Total	3.54 (0.88)	4.05 (0.93)
Belief in a Dangerous World	Democrat	11.55 (3.91)	15.70 (4.16)
	Republican	13.37 (3.93)	14.73 (4.16)
	Total	12.35 (4.01)	15.27 (4.18)
Social Dominance Orientation	Democrat	9.23 (5.00)	19.18 (6.47)
	Republican	9.64 (5.77)	15.25 (6.38)
	Total	9.41 (5.35)	17.46 (6.71)
System Justification	Democrat	14.35 (5.54)	19.26 (4.69)
	Republican	12.08 (6.02)	17.95 (5.26)
	Total	13.34 (5.86)	18.69 (4.98)
Favorability	Democrat	5.34 (1.09)	3.94 (1.35)
	Republican	4.53 (1.24)	5.55 (0.94)
	Total	5.03 (1.24)	4.64 (1.43)

There was a main effect of Facebook condition on individualizing and binding foundations, with higher perceived individualizing foundations for the Democratic profile owner *F*(1, 482) = 32.27, *p* < .001, η_p_^2^ = .063, and higher perceived binding foundations for the Republican profile owner, *F*(1, 482) = 125.10, p < .001, η_p_^2^ = .206. While there was no significant interaction with the participant’s party affiliation with the binding foundation results, there was a significant interaction for the individualizing results, *F*(1, 482) = 35.73, *p* < .001, η_p_^2^ = .069. Specifically, post hoc comparisons revealed that Republican participants did not rate the profiles differently in their endorsement of individualizing foundations (*p* = .843, *d* = -.03), while Democratic participants rated the Democratic profile-owner as more likely to endorse individualizing foundations than the Republican profile-owner (*p* < .001, *d* = 1.02).

As expected, the Republican profile-owner was perceived as having higher NFCC (*F*(1, 482) = 34.38, *p* < .001, η_p_^2^ = .067) , BDW (*F*(1, 482) = 55.85, *p* < .001, η_p_^2^ = .104), SDO (*F*(1, 482) = 208.31, *p* < .001, η_p_^2^ = .302), and SJ (*F*(1, 482) = 119.79, *p* < .001, η_p_^2^ = .199 ) compared to the Democratic profile-owner. Unexpectedly, there was also a main effect of participants’ party affiliation on SDO, *F*(1, 482) = 10.60, *p* = .001, η_p_^2^ = .022, and SJ, *F*(1, 482) = 13.17, *p* < .001, η_p_^2^ = .027 such that Democrats (*M*s = 14.02 & 16.71, *SD*s = 7.61 & 5.70) rated the profile-owner higher than Republicans (*M*s = 12.32 & 14.88, *SD*s = 6.68 & 6.38). And, Facebook condition x participant political party interactions emerged for NFCC (*F*(1, 482) = 6.47, *p* = .011, η_p_^2^ = .013), BDW (*F*(1, 482) = 14.36, *p* < .001, η_p_^2^ = .029), and SDO (*F*(1, 482) = 16.22, *p* < .001, η_p_^2^ = .033) such that Democrats perceived there to be bigger differences between profile-owners than Republicans, consistent with Study 1 and previous research (e.g., Scherer et al., 2015).

The participants’ ratings of the profile-owner’s likeability, intelligence, their overall feelings of positive/negativity towards the profile-owner, the likelihood of becoming friends, and their desire to have the profile-owner as a neighbor were averaged to create a favorability composite (α = .94) where higher numbers indicate greater favorability. An ANOVA revealed a main effect of Facebook condition, *F*(1, 482) = 4.88, *p* = .028, η_p_^2^ = .010 such that the Democratic profile-owners was rated more favorably than the Republican profile-owner. Additionally, a main effect of Participant political party emerged, *F*(1, 482) = 10.81, *p* < .001 η_p_^2^ = .022 such that Democrats (*M* = 4.71, *SD* = 1.43) rated the profile less favorably than Republicans (*M* = 5.01, *SD* = 1.22). These main effects were qualified by a Facebook condition x participant political party interaction, *F*(1, 482) = 137.99, *p* < .001, η_p_^2^ = .223. As expected, Democrat participants rated the Democrat profile more favorably than the Republican profile (*p* < .001, *d* = 1.14), and Republican participants rated the Republican profile more favorably than the Democrat profile (*p* < .001, *d* = .93). In other words, we replicated the typical similarity-liking effect; people preferred the Facebook profile-owner when he was a member of the same political party.

#### Moderated mediation

As in Study 1, we predicted that perceptions of the profile-owner’s moral foundations would mediate the similarity-liking effect, and that the participants’ political party would determine which moral foundation would function as a mediator; however, in Study 2, we were predicting that the effects of political stereotypes about moral foundations would still be significant, even after controlling for a different political stereotypes—stereotypes about traits linked to motivated social cognition. Our moderated mediation analysis was identical to that used in Study 1, with the exception of including the perceived motivated social cognition traits of the profile-owner as additional mediators.

The conditional direct effect of Facebook Profile on the favorability of the profile-owner was significant for Republicans, *B*_*c’*_ = -.27, *SE* = .08, *t*(470) = -3.41, *p* < .001, and marginally significant for Democrats, *B*_*c’*_ = .13, *SE* = .08, *t*(470) = 1.69, *p* = .09. Consistent with the results of Study 1, the conditional indirect effect of Facebook Profile on the profile-owner’s favorability through ratings of *individualizing* foundations was significant only for Democrats (95% CI: 0.11 to 0.33), but not for Republicans (95% CI: -0.01 to 0.02). The index of moderated mediation was significant for individualizing foundations (95% CI: 0.11 to 0.33) with 5,000 bootstrap samples. Also consistent with our predictions and our marginally significant findings in Study 1, the conditional indirect effect of Facebook Profile on favorability through ratings of the profile-owner’s *binding* foundations was significant for Republicans (95% CI: -0.45 to -0.16). It was also significant for Democrats (95% CI: -0.14 to -0.005), but as the significant index of moderated mediation for binding foundations (95% CI: 0.07 to 0.40) suggests, the indirect effect through binding foundations was much stronger for Republicans than Democrats.

All other conditional indirect effects were non-significant barring two exceptions: the conditional indirect effect of Facebook Profile on favorability through ratings of the profile-owner’s SDO was significant for Democratic participants (95% CI: 0.21 to 0.55) and the conditional indirect through NFCC was significant for Republican participants (95% CI: 0.004 to 0.09). Besides the significant moderated mediation indexes for individualizing and binding foundations, the only other significant index of moderated mediation emerged for SDO (95% CI: 0.08 to 0.48). The fact that the moderated mediation indexes for the moral foundations were still significant even after controlling for motivated social cognition variables suggests that political stereotypes about moral foundations can explain the similarity-liking effect above and beyond other political stereotypes.

**Fig 4:**
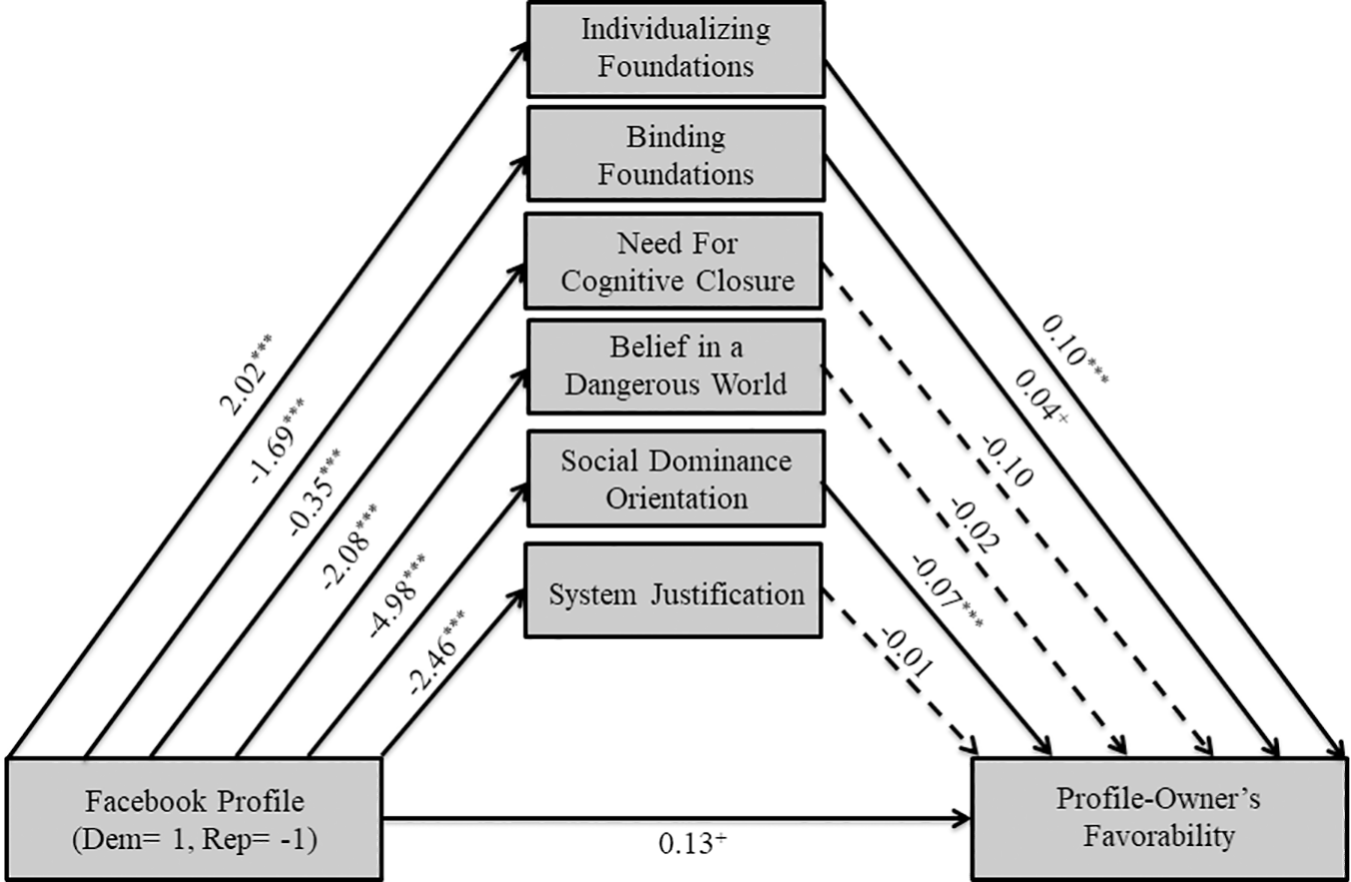
Mediational model for Democrat participants in Study 2. Solid lines indicate paths significant at *p*≤.10, while dashed lines indicate non-significant paths. ^+^ *p*≤.10 *p≤.05 **p≤.01 ***p≤.001.

**Fig 5:**
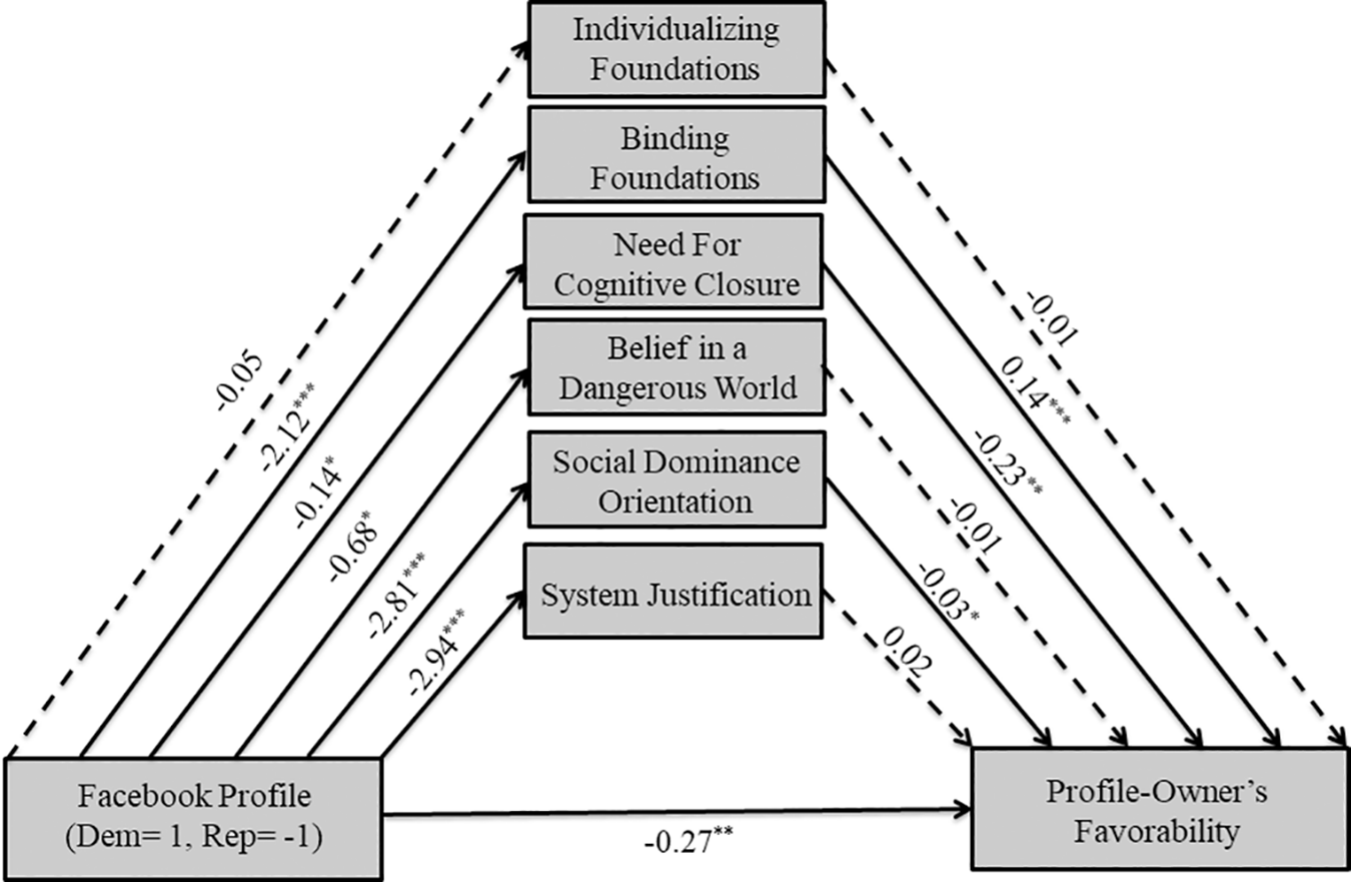
Mediational model for Republican participants in Study 2. Solid lines indicate paths significant at *p*≤.10, while dashed lines indicate non-significant paths. ^+^ *p*≤.10 *p≤.05 **p≤.01 ***p≤.001.

To examine the nature of the moderating effects, as in Study 1, we ran PROCESS Model 4 for Democrats (See Fig 4) and Republicans (See Fig 5) separately. As hypothesized and consistent with Study 1, the Democratic profile-owner was associated with greater perceived endorsement of individualizing foundations for Democratic participants (*B* = 2.02, *SE* = .24, *t*(270) = 8.49, *p* < .001), and lower perceived endorsement of binding foundations (*B* = -1.69, *SE* = .22, *t*(270) = -7.67, *p* < .001), NFCC (*B* = -.35, *SE* = .05, *t*(270) = -6.47, *p* < .001), BDW (*B* = -2.08, *SE* = .24, *t*(270) = -8.50, *p* < .001), SDO (*B* = -4.98, *SE* = .35, *t*(270) = -14.25, *p* < .001), and SJ (*B* = -2.46, *SE* = .31, *t*(270) = -7.86, *p* < .001). Furthermore, greater perceived endorsement of individualizing foundations (*B* = .10, *SE* = .02, *t*(264) = 5.07, *p* < .001) and lesser perceived SDO (*B* = -.07, *SE* = .02, *t*(264) = -4.61, *p* < .001) were associated with more favorability of the profile-owner. Interestingly, greater perceived endorsement of binding foundations was marginally significantly associated with more favorability of the profile-owner (*B* = .04, *SE* = .02, *t*(264) = 1.96, *p* = .05) as was the Democrat profile (*B* = .13, *SE* = .08, *t*(264) = 1.73, *p* = .08), whereas all other predictors were not. In other words, together with the indirect effects reported above, Democrats liked the Democratic profile-owners more partly because they perceived him to endorse more individualizing foundations, even after controlling for the other motivated social cognition variables.

The Republican profile-owner (coded as -1) was not associated with ratings of individualizing foundations (*B* = -.05, *SE* = .25, *t*(212) = -.21, *p* = .84), but, as expected, was associated with greater perceived binding foundations for Republican participants (*B* = -2.12, *SE* = .26, *t*(212) = -8.08, *p* < .001), NFCC (*B* = -.14, *SE* = .06, *t*(212) = -2.16, *p* < .05), BDW (*B* = -.68, *SE* = .28, *t*(212) = -2.46, *p* < .05), SDO (*B* = -2.80, *SE* = .42, *t*(212) = -6.75, *p* < .001), and SJ (*B* = -2.94, *SE* = .39, *t*(212) = -7.56, *p* < .001). Furthermore, the Republican profile (*B* = -.27, *SE* = .08, *t*(206) = -3.30, *p* < .01), along with greater perceived binding foundations (*B* = .14, *SE* = .02, *t*(206) = 5.75, *p* < .001), lesser NFCC (*B* = -.23, *SE* = .08, *t*(206) = -2.69, *p* < .01), and lesser SDO (*B* = -.03, *SE* = .02, *t*(206) = -2.00, *p* < .05) were associated with more favorability, whereas all other predictors were not. In other words, together with the indirect effects reported above, Republican participants liked the Republican profile-owner more in part because they perceived him to endorse binding foundations more, even after controlling for the other motivated social cognition variables. A previous version of this study was run a year earlier with a smaller sample size (N = 164), and results indicated that moderated mediation only occurred for ratings of individualizing foundations and SDO, in that they mediated the effect of Facebook profile on the profile-owner’s favorability only for Democrats, not Republicans. We did not find that binding foundations mediated the effect of Facebook profile on the profile-owner’s favorability for Republicans. We re-ran this study with a suitable sample size (we refrained from combining the studies because of the different timepoints the data were collected); the pattern of results from the study with the smaller sample size is largely the same as from Study 2. See S2 for the moderated mediation analyses for this study.

### Discussion

In Study 2 we again found strong evidence of the similarity-liking effect. Importantly for our hypotheses, we found additional evidence that perceptions of the profile-owner’s moral foundations mediate the similarity-liking effect; specifically, we again see that different kinds of morals matter for Democrats and Republicans when forming impressions. Consistent with Study 1, for Democrats (not Republicans) perceptions of the profile-owner’s individualizing foundations partially mediated the effect of Facebook Profile on the favorability rating of the profile-owner. That is, the similarity-liking effect for Democrats is explained by perceptions of endorsement of individualizing foundations. In Study 2, we also demonstrated that for Republicans, perceptions of the profile-owner’s binding foundations partially mediated the effect of Facebook Profile on the favorability rating of the profile-owner. That is, the similarity-liking effect for Republicans is partially explained by perceptions that the Republican profile-owner was more likely to endorse binding foundations than the Democrat profile owner. And, importantly, we found evidence that moral foundations matter even when controlling for other political stereotypes.

## General discussion

The current experiments provide an important insight into political homophily. Consistent with prior work, we found that people have more positive evaluations of others who share political attitudes with them than those that hold opposing views. Additionally, we replicated previous research demonstrating that people hold political stereotypes about moral foundations and traits linked to motivated social cognition [34, 38]. Previously it has been assumed that the similarity-liking effect in politics was just about political affiliation (i.e., sharing beliefs, or being in an ingroup), but the present work suggests it is not political ideology *per se* that shapes how positively we evaluate another person, but that we view political ideology as a signal for the type of morals the other person endorses. Specifically, we provided evidence that people at least partially evaluate someone else based on the extent to which the other person shares the dominant moral foundations of their own political party, with Democrats privileging individualizing foundations and Republicans binding foundations. Even more striking is that these effects persist when controlling for other political stereotypes. Our results move beyond previous work showing the importance of morality on impression formation [61] by highlighting that the type of morality matters for different political groups.

Interestingly, our results suggest that not only are there political differences in which moral foundations people endorse, but that there are political differences in which moral foundations explain our (moral and non-moral) evaluations of others. This finding may provide insight into one reason that we see political “segregation” on social media [3] or ideological migration, where people move to geographic areas that are consistent with their political ideology [75, 76, 77, 78, 79]. Specifically, people may desire to move to areas that have greater ideological fit because they may view those communities as being more moral.

One curious thing about our samples was that the Republican participants were more likely to endorse individualizing foundations than binding foundations. While some research [48] suggests that Republicans are likely to be high in all five moral foundations, there is no research suggesting that Republicans typically endorse individualizing foundations more than binding. This pattern of responding is more often associated with Democrats. It is possible that in the “post-Trump” era, the moral foundations of Republicans have shifted; for example, it is possible that purity is less important to people who endorse President Trump (who has admitted to sexually assaulting women) than it has been for prior generations of Republicans, but the recent develop of the Moral Foundations Questionnaire prevents examinations of whether the preferred moral foundations of a political party can shift over time.

The current studies provide a foundation for other potential factors to explore to further explain the political similarity-liking effect. While we manipulated the political leanings of the articles, additional features of the article (e.g., how inflammatory the article is; how reliable the article appears to be) that might influence political homophily could also be manipulated. We also chose to utilize the same profile picture across all conditions. Future research could examine how other factors—such as the gender and race of the participant and profile-owner—interact with political ideology. Limitations of our studies include the use of non-representative samples and the use of a single article per condition which introduces the possibility of stimulus sampling issues [80]. Despite these limitations, we believe that the results provide initial evidence for the impact of political stereotypes on homophily, as well as downstream consequences such as willingness to associate with the person, which may help us understand the increasing polarization that is occurring in the U.S. today.

### Conclusions

Our results highlight that people perceive political ideology as a cue to how “moral” a person is, and more importantly, “what kind of” moral a person is, which then influences their evaluations and willingness to associate with that person. Importantly, these studies demonstrate morality is not a singular thing when evaluating others, but that different moral foundations matter for different types of people. Specifically, Democrats are more likely to positively evaluate someone who they believe endorses the individualizing foundations of fairness and preventing harm, and Republicans are more likely to positively evaluate someone who they believe endorses the binding foundations of purity, loyalty, and respect. This process helps explain why people like their political in-group more than their political out-group and provides insights into one contributor of what divides the U.S. politically.

## Supporting information

S1 File Study 1 ANOVA analyses with control group included. (DOCX)

S2 File Study 2a moderated mediation analyses. (DOCX)
