# Supplementary material for: Seeing beyond political affiliations: The mediating role of perceived moral foundations on the partisan similarity-liking effect
Source: PLoS One. 2018 Aug 29;13(8):e0202101. doi: 10.1371/journal.pone.0202101 (PMC6114773; doi:10.1371/journal.pone.0202101)
Supplement: S1 File — (DOCX) [file pone.0202101.s001.docx]

**S1. Study 1 ANOVA Analyses with Control Group Included**

**Profile-owner’s moral foundations**

Ratings of the profile-owner’s individualizing foundations were subjected to a 3 (Facebook profile: Democrat, Control, Republican) x 2 (participant political party: Democrat, Republican) ANOVA.

The effect of participants’ political party was non-significant, *F(*1, 396)=2.62, *p*=.106, η_p_^2^=.007. A main effect of Facebook condition emerged, *F*(2, 396)=4.24, *p*=.015, η_p_^2^=.021. Consistent with previous research (Graham et al., 2012), the Democrat profiles (*M*= 26.13, *SD*= 5.44) were rated as more likely to endorse individualizing foundations than the Republican profiles (*M=*23.67, *SD*= 6.25; *p*=.004, *d*=0.42); neither of the political conditions were rated differently than control (*M*=25.11, *SD*= 5.30); *p*s= .200 & .264, *d*s= 0.19 & 0.25). This main effect was qualified by a participant political party x Facebook condition interaction, *F*(2, 396)=4.22 *p*=.015, η_p_^2^=.021. Simple effects tests revealed that for Democratic participants, there was a significant effect of Facebook condition on the target ratings of individualizing foundations, *F*(2, 265)=10.20, *p*<.001, η_p_^2^=.071, such that the Republican profiles (*M*= 22.44, *SD*= 6.67) were seen as less likely to endorse individualizing foundations than the control profile (*M*= 25.19, *SD*= 5.38; *p*=.005, *d*=-0.45) and Democrat profiles (*M*=26.06, *SD*= 4.78; *p*<.001, *d*=-0.60). For Republicans, the effect of Facebook condition was not significant on the ratings of individualizing foundations. Graham and colleagues (2012) demonstrated that liberals were more likely than conservatives to exaggerate the morals of both in and out-group members, so it is not surprising that Democrats rated the profiles more differently than Republicans.

Ratings of target’s binding foundations were subjected to a 3 (Facebook profile: Democrat, control, Republican) x 2 (participant political party: Republican, Democrat) ANOVA. A main effect of Facebook condition emerged, *F*(2, 396)= 7.80, *p*<.001, η_p_^2^=.038; the Republican profiles (*M*=23.27, *SD*= 4.86) were considered more likely to endorse binding foundations than the control profile (*M*=20.90, *SD*= 4.57; *p*=.005, *d*=0.50), and Democrat profiles (*M*=21.06, *SD*= 5.16; *p*<.001, *d*=0.44). The control and liberal profiles were not rated differently (*p*=.826, *d*=-0.03). The main effect of participants’ political party was marginally significant, *F*(1, 396)=3.31, *p*=.069, η_p_^2^=.008; Republicans (*M*=22.48, *SD*=5.22) were marginally more likely to think the profile-owner would endorse binding foundations than Democrats (*M*=21.56, *SD*=4.92). There was no evidence of a Facebook condition x participant political party interaction, *F*<1.

**Favorability of profile-owner**

The participants’ ratings of the target’s likeability, intelligence, and the likelihood of becoming friends were averaged to create a favorability composite (α=.88) where higher numbers indicate greater favorability. A Facebook condition x participant political party ANOVA revealed a main effect of Facebook condition, *F*(4, 489)=11.35, *p*<.001, η_p_^2^=.124. The Republican profile-owners (*M*=4.46, *SD*= 1.39) were rated marginally less favorably than the Democrat profile-owners (*M*=5.00, *SD*= 1.19; *p*=.065, *d*=0.42). The control profile-owner (*M*=5.24, *SD*= 0.97) was rated more favorably than both the Democrat profile-owner (*p*=.015, *d*=0.22) and the Republican profile-owner (*p*<.001, *d*=0.65).

A Facebook condition x political party interaction also emerged, *F*(2, 396)=23.26, *p*<.001, η_p_^2^=.105. For Democrat participants, there was a simple effect of Facebook condition, *F*(2, 265)= 34.05, *p*<.001, η_p_^2^=.204. Democrats rated the Republican profile-owner less favorably than other profiles (M= 4.06, SD= 1.36; *p*s<.001, *d*s= -0.98 to -1.02); they did not rate the control (*M*=5.19, *SD*= 0.92) and Democrat profile-owners differently (M= 5.25, SD= 0.97; *p*=.712, *d*=0.06). There was also a significant simple effect of Facebook condition for Republican participants, *F*(2, 131)=6.07, *p*=.003, η_p_^2^=.085. Republicans rated the Democrat profile-owners (*M*=4.49, *SD*= 1.41) less favorably than the other profiles (*p*s<.001, *d*s= -0.54 to -0.70). Republicans did not rate the control (*M*=5.37, *SD*= 1.08) and Republican (*M*=5.18, *SD*= 1.13) profile-owners differently (*p*=.516, *d*=0.17).
