# Supplementary material for: Seeing beyond political affiliations: The mediating role of perceived moral foundations on the partisan similarity-liking effect
Source: PLoS One. 2018 Aug 29;13(8):e0202101. doi: 10.1371/journal.pone.0202101 (PMC6114773; doi:10.1371/journal.pone.0202101)
Supplement: S2 File — (DOCX) [file pone.0202101.s002.docx]

**S2. Study 2a Moderated Mediation Analyses**

**Matrix**

Run MATRIX procedure:

************* PROCESS Procedure for SPSS Release 2.16.3 ******************

Written by Andrew F. Hayes, Ph.D. www.afhayes.com

Documentation available in Hayes (2013). www.guilford.com/p/hayes3

**************************************************************************

Model = 59

Y = Favorability

X = facebook

M1 = Target_Individualizing

M2 = Target_Binding

M3 = tarBDW

M4 = tarNFC

M5 = tarSDO

M6 = tarSJ

W = Particant_Political_Party

Sample size

164

**************************************************************************

Outcome: Target_Individualizing

Model Summary

R R-sq MSE F df1 df2 p

.4338 .1882 12.5944 12.3638 3.0000 160.0000 .0000

Model

coeff se t p LLCI ULCI

constant 17.9216 .2808 63.8149 .0000 17.3670 18.4762

facebook 1.0129 .2808 3.6067 .0004 .4583 1.5675

Particant_Political_Party .1921 .2808 .6842 .4949 -.3625 .7468

int_1 1.1897 .2808 4.2363 .0000 .6351 1.7443

Product terms key:

int_1 facebook X Particant_Political_Party

**************************************************************************

Outcome: Target_Binding

Model Summary

R R-sq MSE F df1 df2 p

.4728 .2235 15.2646 15.3511 3.0000 160.0000 .0000

Model

coeff se t p LLCI ULCI

constant 15.3726 .3092 49.7209 .0000 14.7620 15.9832

facebook -2.0789 .3092 -6.7240 .0000 -2.6895 -1.4683

Particant_Political_Party .2183 .3092 .7059 .4813 -.3923 .8289

int_1 .2772 .3092 .8965 .3713 -.3334 .8878

Product terms key:

int_1 facebook X Particant_Political_Party

**************************************************************************

Outcome: tarBDW

Model Summary

R R-sq MSE F df1 df2 p

.3626 .1315 19.2156 8.0743 3.0000 160.0000 .0000

Model

coeff se t p LLCI ULCI

constant 13.8812 .3469 40.0161 .0000 13.1961 14.5663

facebook -1.5941 .3469 -4.5955 .0000 -2.2792 -.9091

Particant_Political_Party -.5324 .3469 -1.5349 .1268 -1.2175 .1526

int_1 -.1015 .3469 -.2927 .7701 -.7866 .5835

Product terms key:

int_1 facebook X Particant_Political_Party

**************************************************************************

Outcome: tarNFC

Model Summary

R R-sq MSE F df1 df2 p

.2345 .0550 18.3789 3.1048 3.0000 160.0000 .0282

Model

coeff se t p LLCI ULCI

constant 18.5276 .3393 54.6127 .0000 17.8576 19.1976

facebook -.7360 .3393 -2.1693 .0315 -1.4060 -.0660

Particant_Political_Party .7044 .3393 2.0763 .0395 .0344 1.3744

int_1 .0346 .3393 .1020 .9189 -.6354 .7046

Product terms key:

int_1 facebook X Particant_Political_Party

**************************************************************************

Outcome: tarSDO

Model Summary

R R-sq MSE F df1 df2 p

.6262 .3921 29.8229 34.3955 3.0000 160.0000 .0000

Model

coeff se t p LLCI ULCI

constant 12.7226 .4322 29.4398 .0000 11.8691 13.5761

facebook -4.1552 .4322 -9.6150 .0000 -5.0087 -3.3017

Particant_Political_Party .1042 .4322 .2410 .8098 -.7493 .9576

int_1 -.7736 .4322 -1.7901 .0753 -1.6271 .0799

Product terms key:

int_1 facebook X Particant_Political_Party

**************************************************************************

Outcome: tarSJ

Model Summary

R R-sq MSE F df1 df2 p

.5301 .2810 22.7982 20.8455 3.0000 160.0000 .0000

Model

coeff se t p LLCI ULCI

constant 15.5098 .3778 41.0479 .0000 14.7636 16.2560

facebook -2.3791 .3778 -6.2964 .0000 -3.1253 -1.6328

Particant_Political_Party 1.6546 .3778 4.3789 .0000 .9084 2.4008

int_1 .7657 .3778 2.0264 .0444 .0195 1.5119

Product terms key:

int_1 facebook X Particant_Political_Party

**************************************************************************

Outcome: Favorability

Model Summary

R R-sq MSE F df1 df2 p

.7141 .5099 .8688 10.2663 15.0000 148.0000 .0000

Model

coeff se t p LLCI ULCI

constant 3.0429 .6480 4.6955 .0000 1.7623 4.3235

Target_Individualizing .0893 .0254 3.5147 .0006 .0391 .1395

Target_Binding .0114 .0238 .4796 .6322 -.0355 .0583

tarBDW -.0013 .0208 -.0640 .9491 -.0424 .0398

tarNFC .0185 .0197 .9411 .3482 -.0204 .0574

tarSDO -.0285 .0184 -1.5456 .1243 -.0649 .0079

tarSJ .0086 .0187 .4595 .6465 -.0284 .0455

facebook -.2182 .1020 -2.1385 .0341 -.4198 -.0166

int_2 .0357 .0254 1.4056 .1619 -.0145 .0859

int_3 -.0476 .0238 -2.0047 .0468 -.0945 -.0007

int_4 .0053 .0208 .2544 .7996 -.0358 .0464

int_5 .0212 .0197 1.0748 .2842 -.0177 .0600

int_6 -.0499 .0184 -2.7094 .0075 -.0863 -.0135

int_7 -.0049 .0187 -.2628 .7931 -.0419 .0320

Particant_Political_Party .3030 .6480 .4675 .6408 -.9776 1.5836

int_8 .2245 .1020 2.2003 .0293 .0229 .4261

Product terms key:

int_2 Target_Individualizing X Particant_Political_Party

int_3 Target_Binding X Particant_Political_Party

int_4 tarBDW X Particant_Political_Party

int_5 tarNFC X Particant_Political_Party

int_6 tarSDO X Particant_Political_Party

int_7 tarSJ X Particant_Political_Party

int_8 facebook X Particant_Political_Party

******************** DIRECT AND INDIRECT EFFECTS *************************

Conditional direct effect(s) of X on Y at values of the moderator(s):

Particant_Political_Party Effect SE t p LLCI ULCI

-1.0000 -.4427 .1479 -2.9924 .0032 -.7351 -.1503

1.0000 .0063 .1406 .0449 .9643 -.2715 .2841

Conditional indirect effect(s) of X on Y at values of the moderator(s):

Mediator

Particant_Political_Party Effect Boot SE BootLLCI BootULCI

Target_Individualizing -1.0000 -.0095 .0328 -.1142 .0347

Target_Individualizing 1.0000 .2754 .0892 .1115 .4598

Mediator

Particant_Political_Party Effect Boot SE BootLLCI BootULCI

Target_Binding -1.0000 -.1390 .1028 -.3579 .0558

Target_Binding 1.0000 .0653 .0567 -.0274 .2034

Mediator

Particant_Political_Party Effect Boot SE BootLLCI BootULCI

tarBDW -1.0000 .0099 .0438 -.0834 .0939

tarBDW 1.0000 -.0067 .0565 -.1255 .1064

Mediator

Particant_Political_Party Effect Boot SE BootLLCI BootULCI

tarNFC -1.0000 .0020 .0266 -.0447 .0711

tarNFC 1.0000 -.0278 .0318 -.1316 .0077

Mediator

Particant_Political_Party Effect Boot SE BootLLCI BootULCI

tarSDO -1.0000 -.0725 .1041 -.2777 .1404

tarSDO 1.0000 .3863 .1515 .1117 .7095

Mediator

Particant_Political_Party Effect Boot SE BootLLCI BootULCI

tarSJ -1.0000 -.0425 .1044 -.2617 .1510

tarSJ 1.0000 -.0059 .0404 -.1034 .0627

******************** INDEX OF MODERATED MEDIATION ************************

Mediator

Index SE(Boot) BootLLCI BootULCI

Target_Individualizing .2849 .0957 .1106 .5003

Target_Binding .2043 .1174 -.0036 .4591

tarBDW -.0166 .0712 -.1658 .1265

tarNFC -.0299 .0410 -.1405 .0336

tarSDO .4588 .1827 .1163 .8332

tarSJ .0365 .1124 -.1767 .2601

When the moderator is dichotomous, this is a test of equality of the

conditional indirect effects in the two groups.

******************** ANALYSIS NOTES AND WARNINGS *************************

Number of bootstrap samples for bias corrected bootstrap confidence intervals:

5000

Level of confidence for all confidence intervals in output:

95.00

------ END MATRIX -----

restore.

USE ALL.

COMPUTE filter_$=(Particant_Political_Party = 1).

VARIABLE LABELS filter_$ 'Particant_Political_Party = 1 (FILTER)'.

VALUE LABELS filter_$ 0 'Not Selected' 1 'Selected'.

FORMATS filter_$ (f1.0).

FILTER BY filter_$.

EXECUTE.

/* PROCESS for SPSS 2.16.3 */.

/* Written by Andrew F. Hayes */.

/* www.afhayes.com */.

/* Copyright 2012-2016 */.

/* Online distribution other than through */.

/* www.afhayes.com or processmacro.org is not authorized */.

/* Please read the documentation */.

/* available in Appendix A of */.

/* Hayes (2013) prior to use */.

/* www.guilford.com/p/hayes3 */.

/* Documentation available in Appendix A of http://www.guilford.com/p/hayes3 */.

preserve.

set printback=off.

**Matrix**

Run MATRIX procedure:

************* PROCESS Procedure for SPSS Release 2.16.3 ******************

Written by Andrew F. Hayes, Ph.D. www.afhayes.com

Documentation available in Hayes (2013). www.guilford.com/p/hayes3

**************************************************************************

Model = 4

Y = Favorability

X = facebook

M1 = Target_Individualizing

M2 = Target_Binding

M3 = tarBDW

M4 = tarNFC

M5 = tarSDO

M6 = tarSJ

Sample size

94

**************************************************************************

Outcome: Target_Individualizing

Model Summary

R R-sq MSE F df1 df2 p

.5809 .3374 9.7173 46.8457 1.0000 92.0000 .0000

Model

coeff se t p LLCI ULCI

constant 18.1137 .3218 56.2866 .0000 17.4746 18.7529

facebook 2.2026 .3218 6.8444 .0000 1.5635 2.8418

**************************************************************************

Outcome: Target_Binding

Model Summary

R R-sq MSE F df1 df2 p

.4352 .1894 14.1666 21.5010 1.0000 92.0000 .0000

Model

coeff se t p LLCI ULCI

constant 15.5909 .3886 40.1243 .0000 14.8191 16.3626

facebook -1.8017 .3886 -4.6369 .0000 -2.5735 -1.0300

**************************************************************************

Outcome: tarBDW

Model Summary

R R-sq MSE F df1 df2 p

.3604 .1299 19.6414 13.7360 1.0000 92.0000 .0004

Model

coeff se t p LLCI ULCI

constant 13.3488 .4575 29.1759 .0000 12.4401 14.2574

facebook -1.6957 .4575 -3.7062 .0004 -2.6044 -.7870

**************************************************************************

Outcome: tarNFC

Model Summary

R R-sq MSE F df1 df2 p

.1755 .0308 15.7935 2.9224 1.0000 92.0000 .0907

Model

coeff se t p LLCI ULCI

constant 19.2320 .4103 46.8764 .0000 18.4171 20.0468

facebook -.7014 .4103 -1.7095 .0907 -1.5162 .1135

**************************************************************************

Outcome: tarSDO

Model Summary

R R-sq MSE F df1 df2 p

.6574 .4322 32.5522 70.0233 1.0000 92.0000 .0000

Model

coeff se t p LLCI ULCI

constant 12.8268 .5890 21.7770 .0000 11.6569 13.9966

facebook -4.9288 .5890 -8.3680 .0000 -6.0986 -3.7590

**************************************************************************

Outcome: tarSJ

Model Summary

R R-sq MSE F df1 df2 p

.3239 .1049 22.6511 10.7826 1.0000 92.0000 .0014

Model

coeff se t p LLCI ULCI

constant 17.1644 .4913 34.9345 .0000 16.1886 18.1402

facebook -1.6134 .4913 -3.2837 .0014 -2.5892 -.6376

**************************************************************************

Outcome: Favorability

Model Summary

R R-sq MSE F df1 df2 p

.7576 .5739 .7653 16.5492 7.0000 86.0000 .0000

Model

coeff se t p LLCI ULCI

constant 3.3459 .8583 3.8984 .0002 1.6397 5.0520

Target_Individualizing .1250 .0352 3.5483 .0006 .0550 .1951

Target_Binding -.0362 .0272 -1.3293 .1873 -.0904 .0179

tarBDW .0040 .0266 .1486 .8822 -.0490 .0569

tarNFC .0397 .0275 1.4416 .1530 -.0150 .0944

tarSDO -.0784 .0224 -3.5033 .0007 -.1229 -.0339

tarSJ .0037 .0213 .1727 .8633 -.0386 .0460

facebook .0063 .1319 .0478 .9620 -.2560 .2686

******************** DIRECT AND INDIRECT EFFECTS *************************

Direct effect of X on Y

Effect SE t p LLCI ULCI

.0063 .1319 .0478 .9620 -.2560 .2686

Indirect effect of X on Y

Effect Boot SE BootLLCI BootULCI

TOTAL .6865 .1354 .4446 .9758

Target_Individualizing .2754 .0875 .1151 .4597

Target_Binding .0653 .0581 -.0260 .2116

tarBDW -.0067 .0560 -.1211 .1066

tarNFC -.0278 .0302 -.1202 .0077

tarSDO .3863 .1510 .1110 .7158

tarSJ -.0059 .0394 -.0944 .0664

******************** ANALYSIS NOTES AND WARNINGS *************************

Number of bootstrap samples for bias corrected bootstrap confidence intervals:

5000

Level of confidence for all confidence intervals in output:

95.00

------ END MATRIX -----

restore.

USE ALL.

COMPUTE filter_$=(Particant_Political_Party = -1).

VARIABLE LABELS filter_$ 'Particant_Political_Party = -1 (FILTER)'.

VALUE LABELS filter_$ 0 'Not Selected' 1 'Selected'.

FORMATS filter_$ (f1.0).

FILTER BY filter_$.

EXECUTE.

/* PROCESS for SPSS 2.16.3 */.

/* Written by Andrew F. Hayes */.

/* www.afhayes.com */.

/* Copyright 2012-2016 */.

/* Online distribution other than through */.

/* www.afhayes.com or processmacro.org is not authorized */.

/* Please read the documentation */.

/* available in Appendix A of */.

/* Hayes (2013) prior to use */.

/* www.guilford.com/p/hayes3 */.

/* Documentation available in Appendix A of http://www.guilford.com/p/hayes3 */.

preserve.

set printback=off.

**Matrix**

Run MATRIX procedure:

************* PROCESS Procedure for SPSS Release 2.16.3 ******************

Written by Andrew F. Hayes, Ph.D. www.afhayes.com

Documentation available in Hayes (2013). www.guilford.com/p/hayes3

**************************************************************************

Model = 4

Y = Favorability

X = facebook

M1 = Target_Individualizing

M2 = Target_Binding

M3 = tarBDW

M4 = tarNFC

M5 = tarSDO

M6 = tarSJ

Sample size

70

**************************************************************************

Outcome: Target_Individualizing

Model Summary

R R-sq MSE F df1 df2 p

.0440 .0019 16.4870 .1318 1.0000 68.0000 .7177

Model

coeff se t p LLCI ULCI

constant 17.7294 .4871 36.3976 .0000 16.7574 18.7014

facebook -.1768 .4871 -.3630 .7177 -1.1488 .7952

**************************************************************************

Outcome: Target_Binding

Model Summary

R R-sq MSE F df1 df2 p

.5030 .2530 16.7501 23.0282 1.0000 68.0000 .0000

Model

coeff se t p LLCI ULCI

constant 15.1543 .4910 30.8657 .0000 14.1746 16.1341

facebook -2.3561 .4910 -4.7988 .0000 -3.3358 -1.3764

**************************************************************************

Outcome: tarBDW

Model Summary

R R-sq MSE F df1 df2 p

.3299 .1088 18.6394 8.3052 1.0000 68.0000 .0053

Model

coeff se t p LLCI ULCI

constant 14.4137 .5179 27.8295 .0000 13.3801 15.4472

facebook -1.4926 .5179 -2.8819 .0053 -2.5261 -.4591

**************************************************************************

Outcome: tarNFC

Model Summary

R R-sq MSE F df1 df2 p

.1643 .0270 21.8767 1.8859 1.0000 68.0000 .1742

Model

coeff se t p LLCI ULCI

constant 17.8232 .5611 31.7645 .0000 16.7035 18.9429

facebook -.7706 .5611 -1.3733 .1742 -1.8902 .3491

**************************************************************************

Outcome: tarSDO

Model Summary

R R-sq MSE F df1 df2 p

.5559 .3090 26.1304 30.4080 1.0000 68.0000 .0000

Model

coeff se t p LLCI ULCI

constant 12.6184 .6132 20.5769 .0000 11.3947 13.8421

facebook -3.3816 .6132 -5.5143 .0000 -4.6053 -2.1579

**************************************************************************

Outcome: tarSJ

Model Summary

R R-sq MSE F df1 df2 p

.5525 .3053 22.9973 29.8805 1.0000 68.0000 .0000

Model

coeff se t p LLCI ULCI

constant 13.8553 .5753 24.0838 .0000 12.7073 15.0033

facebook -3.1447 .5753 -5.4663 .0000 -4.2927 -1.9967

**************************************************************************

Outcome: Favorability

Model Summary

R R-sq MSE F df1 df2 p

.6447 .4157 1.0122 6.3015 7.0000 62.0000 .0000

Model

coeff se t p LLCI ULCI

constant 2.7399 .9915 2.7635 .0075 .7580 4.7218

Target_Individualizing .0536 .0370 1.4494 .1523 -.0203 .1275

Target_Binding .0590 .0406 1.4539 .1510 -.0221 .1401

tarBDW -.0066 .0328 -.2018 .8407 -.0722 .0590

tarNFC -.0026 .0283 -.0928 .9263 -.0593 .0540

tarSDO .0214 .0303 .7070 .4822 -.0392 .0820

tarSJ .0135 .0321 .4209 .6753 -.0506 .0776

facebook -.4427 .1597 -2.7722 .0073 -.7619 -.1235

******************** DIRECT AND INDIRECT EFFECTS *************************

Direct effect of X on Y

Effect SE t p LLCI ULCI

-.4427 .1597 -2.7722 .0073 -.7619 -.1235

Indirect effect of X on Y

Effect Boot SE BootLLCI BootULCI

TOTAL -.2515 .1382 -.5714 -.0210

Target_Individualizing -.0095 .0319 -.1026 .0359

Target_Binding -.1390 .0994 -.3644 .0415

tarBDW .0099 .0436 -.0798 .0993

tarNFC .0020 .0261 -.0432 .0706

tarSDO -.0725 .1034 -.2720 .1389

tarSJ -.0425 .1030 -.2596 .1473

******************** ANALYSIS NOTES AND WARNINGS *************************

Number of bootstrap samples for bias corrected bootstrap confidence intervals:

5000

Level of confidence for all confidence intervals in output:

95.00

------ END MATRIX -----
